# Current Challenges for IDO2 as Target in Cancer Immunotherapy

**DOI:** 10.3389/fimmu.2021.679953

**Published:** 2021-04-21

**Authors:** Giada Mondanelli, Martina Mandarano, Maria Laura Belladonna, Chiara Suvieri, Cristina Pelliccia, Guido Bellezza, Angelo Sidoni, Agostinho Carvalho, Ursula Grohmann, Claudia Volpi

**Affiliations:** ^1^ Department of Medicine and Surgery, Section of Pharmacology, University of Perugia, Perugia, Italy; ^2^ Department of Medicine and Surgery, Section of Anatomic Pathology and Histology, University of Perugia, Perugia, Italy; ^3^ Life and Health Sciences Research Institute (ICVS), School of Medicine, University of Minho, Braga, Portugal; ^4^ ICVS/3B’s - PT Government Associate Laboratory, Braga/Guimarães, Portugal

**Keywords:** IDO2, tryptophan metabolism, pseudoenzymes, NSCLC, PDAC

## Abstract

Immune checkpoint inhibitors have revolutionized the clinical approach of untreatable tumors and brought a breath of fresh air in cancer immunotherapy. However, the therapeutic effects of these drugs only cover a minority of patients and alternative immunotherapeutic targets are required. Metabolism of l-tryptophan (Trp) *via* the kynurenine pathway represents an important immune checkpoint mechanism that controls adaptive immunity and dampens exaggerated inflammation. Indoleamine 2,3-dioxygenase 1 (IDO1), the enzyme catalyzing the first, rate–limiting step of the pathway, is expressed in several human tumors and IDO1 catalytic inhibitors have reached phase III clinical trials, unfortunately with disappointing results. Although much less studied, the IDO1 paralog IDO2 may represent a valid alternative as drug target in cancer immunotherapy. Accumulating evidence indicates that IDO2 is much less effective than IDO1 in metabolizing Trp and its functions are rather the consequence of interaction with other, still undefined proteins that may vary in distinct inflammatory and neoplastic contexts. As a matter of fact, the expression of *IDO2* gene variants is protective in PDAC but increases the risk of developing tumor in NSCLC patients. Therefore, the definition of the IDO2 interactome and function in distinct neoplasia may open innovative avenues of therapeutic interventions.

## Introduction

Over the course of evolution, the metabolism of l-tryptophan (Trp), an essential amino acid for mammals, has evolved to be a primary control node in the regulation of immune responses (1). In this regard, the most important enzyme is indoleamine 2,3-dioxygenase 1 (IDO1), a monomeric, heme-containing enzyme that catalyzes the initial, rate-limiting step in the degradation of Trp along the so-called kynurenine pathway (2, 3). l-kynurenine (Kyn), the first product of this pathway, promotes immunoregulatory effects *via* activation of the aryl hydrocarbon receptor (AhR) in dendritic cells (DCs) and T lymphocytes (4–7). By degrading Trp, IDO1 also depletes the essential amino acid in microenvironments, thus activating the general control non-depressible 2 (GCN2) kinase pathway and the dysfunction of T cells (8, 9). In addition to catalytic activity, IDO1 is endowed with a signaling function that, upon tyrosine phosphorylation of immune tyrosine inhibitory motifs (ITIMs) in the small noncatalytic domain of the enzyme, allows the direct interaction with Src homology region 2 domain-containing phosphatase-1 and -2 (SHP-1 and SHP-2, respectively) and confers long-term immunoregulatory properties on DCs (10, 11). The same domain also contains a YxxM motif that, once tyrosine phosphorylated, binds the p85 subunit of class I phosphoinositide 3-kinases (PI3Ks) that drive IDO1 trafficking from cytosol (where exerts the catalytic function) to early endosomes, thus favoring IDO1 signaling activity (12).

IDO1 is expressed in several human tumors and immune cells infiltrating the tumor mass (13) and, for this reason, IDO1 catalytic inhibitors have been used as experimental drugs in cancer immunotherapy (9, 14). One of these inhibitors, epacadostat, was recently coadministered with pembrolizumab, an immune checkpoint inhibitor, in patients with unresectable or mestastatic melanoma in a phase III trial. However, the results were disappointing, as epacadostat did not show the efficacy observed in the previous phase II trial (15). Several causes may have determined this failure, including an inadequate selection of patients. However, considering its complex functional dynamics as described above, IDO1 may represent a hard molecule to be exploited as an effective drug target.

Some years ago, a paralog of IDO1, i.e., IDO2, was discovered. In accordance with those studies, the *IDO1* gene (expressed in mammals and fungi) derived from the duplication of *IDO2* (expressed in all organisms, including bacteria), thus considered more ancestral than IDO1, and the two genes can be detected in tandem in chromosome 8 in both humans and rodents (16). Although it can also initiate the kynurenine pathway, IDO2 affinity for the Trp substrate and catalytic efficacy in producing Kyn are very low or almost negligible (17). Therefore, IDO2 may contribute only a minimal role to overall Trp metabolism (18). Nevertheless, IDO2 is expressed at high levels in some human tumors and, therefore, understanding its true function/s in neoplastic contexts may propel the development of new drugs targeting enzymes of the kynurenine pathway in cancer immunotherapy.

## The “Mystery” of IDO2 Function: Hints From Autoimmune/Chronic Inflammatory Diseases

Since the discovery of IDO2, the primary efforts of the scientific community were addressed to deciphering its role in the modulation of immune responses, assuming that a remarkable sequence homology with IDO1 was accompanied by a parallel analogy in the immunoregulatory functions. A first attempt to discern the physiological and pathophysiological functions of IDO2 was made through the generation of mice deficient in the *Ido2* gene. The characterization of these genetically deficient mice highlighted that IDO1 and IDO2 show some important differences. As a matter of fact, the ablation of IDO2 did not affect Kyn circulating levels, suggesting a specific role for IDO2 and distinct from the enzymatic function. IDO2 was essential for IDO1-dependent induction of T regulatory (Treg) cells and, in IDO1 knockout mice, a great amount of *Ido2* transcripts were subjected to alternative splicing, implying a mutual influence between the two paralogues regarding their expression and function (19). Nevertheless, in a classical model of hapten-induced contact hypersensitivity (CHS), the contribution of IDO2 in the adaptive inflammatory response was remarkably different from that of IDO1, with a reduced response and a significant impairment in proinflammatory cytokines production in IDO2-deficient mice (19).

The positive role of IDO2 in the development of inflammatory processes was further and elegantly demonstrated by means of a murine model of autoimmune arthritis, i.e., the KRN.g7 mice, genetically deficient for either the *Ido1* or *Ido2* gene, which revealed that IDO2, but not IDO1, is necessary for arthritis development. IDO2-deficient mice showed a delayed onset and reduced arthritis severity, due to a reduction in pathogenic antibody-secreting cells and corresponding decrease in autoantibodies (20). A thorough analysis of the IDO2 involvement in the pathogenesis of arthritis revealed that IDO2 participates in the initiation stage of the response prior to the generation of autoantibodies; however, no clues for the exact mechanism of action of IDO2 could be obtained. In the same experimental setting, the specific silencing of IDO2 in B cells significantly reduced total arthritis severity, confirming the role of IDO2 in disease initiation and progression and pinpointing IDO2 as an innovative target for the treatment of this autoimmune disease (21). Again, no evidence emerged from these studies revealing the molecular mechanism of IDO2 in regulating the autoimmune response in the arthritis model. In line with these observations, in humans, the expression of an IDO2 variant lacking catalytic activity is associated with reduced risk of Crohn’s disease (7).

In contrast with these studies, in a model of psoriasis-like inflammation, the manifestations of the disease were significantly worse in the IDO2 KO mice (22). In fact, full active IDO2 was endowed with the ability to control the production of pro-inflammatory IL-17, thus contributing to the suppression of skin inflammation. Therefore, the results obtained in the murine model of psoriasis add more complexity than reinforcing the hypothesis of a proinflammatory role of IDO2.

A perspective that could reconcile the apparent divergence of the results obtained in different experimental models of inflammation/autoimmunity is that the activity of IDO2 may be strictly related to the physiopathologic context and cellular microenvironment. In support of this hypothesis, a recent study revealed that, in two different cohorts of patients with aspergillosis, specific and different patterns of *IDO2* single nucleotide polymorphisms (SNPs) can be observed. More specifically, in patients with cystic fibrosis, SNPs that profoundly affect IDO2 expression and/or function did not associate with an increased risk of aspergillosis, whereas the same SNPs were required for optimal antifungal activity in patients who have undergone hematopoietic stem cell transplantation (23).

## IDO2 Expression and Function in Tumors: A Matter of Genetics

In general, overexpression of IDO2 in tumors appears to be less frequent than IDO1 (13). More recently, human gastric, colorectal, and renal carcinomas have been found to constitutively express both IDO1 and IDO2 (24), and the same has been observed in brain tumors (25). Interestingly, IDO2 is particularly overexpressed in pancreatic ductal adenocarcinoma (PDAC) (26) and non-small-cell lung cancer (NSCLC) (27).

A unique feature of the *IDO2* gene in humans is the high prevalence of two inactivating SNPs, which allow the opportunity to carry out loss-of-function studies directly in humans and to compare patients’ data with those from *Ido2^−/−^* and *Ido2^+/+^* tumor-bearing mice. These SNPs are rs10109853, which leads to a > 90% reduction in IDO2 catalytic activity (R248W), and rs4503083, which generates a premature stop codon (Y359X) and completely inactivates IDO2 activity (26). Large scale sequencing analysis revealed that these two nonfunctional alleles of IDO2 are frequently distributed in human populations of Asian, European, and African descent. However, although both of these SNPs are highly prevalent in human populations, their clinical significance has remained unclear.

### IDO2 in Mouse Tumors

Similarly to human cancer, IDO2 is not frequently expressed in mouse tumors. However, the opportunity to use *Ido2^−/−^* mice as compared to IDO2-expressing counterparts allows the study of the function of endogenous IDO2 in tumor-bearing individuals.

Lewis lung carcinoma (LLC), isolated from a spontaneous epidermoid carcinoma of the mouse lung, does not express IDO1 and IDO2. Therefore, when injecting LLC cells into IDO2 KO mice, no IDO2 will be present anywhere in the organism. In these conditions, tumor growth is suppressed, IFN-γ secretion is enhanced in the tumor bed, and the number of CD8^+^ tumor infiltrating lymphocytes (TILs) is increased (28).

Endogenous IDO2 may also be involved in mechanisms of tumorigenesis. To investigate this possibility, Nevler et al. (29) used the KC transgenic mouse model (30) in which an inducible oncogenic *Kras* allele is activated in pancreatic progenitor cells, thus leading to the development of ductal lesions that recapitulate the full spectrum of human pancreatic intraepithelial neoplasias, putative precursors to invasive pancreatic cancer (PDAC). They found that PDAC development was significantly decreased when *Ido2^−/−^* alleles had been introduced into the KC strain *via* interbreeding. No major changes could be observed for immune populations infiltrating the tumor. Unexpectedly, the impact of IDO2 loss in tumor growth was mainly associated with females. In fact, no tumor development at all could be observed in *Ido2^−/−^* females under study (29).

B16/BL6 melanoma is an example of mouse tumor cells that express IDO2 (31). In order to understand the role of the enzyme in this tumor, Liu et al. performed *Ido2* gene silencing *in vitro via* small interfering RNA (siRNA). Reduction of IDO2 expression in B16/BL6 cells inhibited cancer cell proliferation, arrest of the cell cycle in G1, increased the rate of apoptosis, and reduced cell migration. These *in vitro* effects were accompanied by a decrease in NAD^+^ (a metabolite downstream the kynurenine pathway). Addition of exogenous NAD^+^ to B16/BL6 cell cultures weakened the effect of IDO2 downregulation. *In vivo*, B16/BL6 cells with reduced IDO2 expression grew less than IDO2-competent cells (31). The possible involvement of immune cells and of endogenous IDO2 was not addressed in this study.

Thus, as a whole, the available data would indicate that IDO2, either endogenous or expressed by the tumor, exerts immunosuppressive and pro-tumor effects in mouse models of cancer.

### IDO2 in Human Pancreatic Ductal Adenocarcinoma (PDAC)

PDAC is one of the most aggressive and lethal diseases. Less than 10% of patients with PDAC has a life expectance of five years after diagnosis (32). Despite encouraging evidence for other tumors, including non-small-cell lung cancer (NSCLC; see below), the use of immune checkpoint inhibitors, such as anti-CTLA4 (ipilimumab and tremelimumab) and anti-PD1 (nivolumab and pembrolizumab) antibodies, has shown poor efficacy in PDAC as monotherapy. Although clinical trials are undergoing with combinations of two immune checkpoint inhibitors or one immune checkpoint inhibitor and chemotherapy, the road to an effective immunotherapeutic cure for PDAC appears full of obstacles (33).

IDO2 may represent an important drug target in PDAC therapy. In fact, IDO2 is frequently upregulated in human PDAC (29). In a recent study, the analysis of the prevalence of the two *IDO2*-inactivating SNPs together with the treatment outcomes indicated that, in PDAC patients having received adjuvant radiotherapy, the “*IDO2*-deficient status” significantly associates with improved disease-free survival (29). Therefore, the *IDO2* genotype has the immediate potential to influence the PDAC care decision-making process through stratification of those patients who stand to benefit from adjuvant radiotherapy.

An additional interesting aspect of IDO2 involvement in PDAC is sexual dimorphism. In fact, along the same line of *Ido2^−/−^* female mice in which development of PDAC is significantly less than the male counterparts, female patients with PDAC rarely harbor the *IDO2*-deficient status (29). Therefore, these data would suggest that female patients with PDAC should be taken into high consideration for immunotherapy involving IDO2 inhibition.

### IDO2 in Human Non-Small Cell Lung Cancer (NSCLC)

NSCLC, representing the majority (approximately 85%) of lung malignancies (34), is in general insensitive to standard treatments with chemotherapeutic drugs. Therapy of NSCLC has been partly improved by the use of nivolumab. In fact, nivolumab treatment has been associated with longer overall survival than the chemotherapeutic docetaxel among patients with previously treated NSCLC, regardless of PD-L1 levels (35, 36). Nevertheless, the response rate was not more than 20%. A more recent study (37) indicated that, in patients with untreated stage IV or recurrent NSCLC with a PD-L1 expression level of 5% or more, nivolumab was not associated with longer progression-free survival than chemotherapy. Therefore, although more therapeutic options are available than PDAC, alternative drug targets are also needed in NSLC.

In a recent study with 191 NSCLC patients, IDO2 was highly expressed in 84% of samples and its expression was strictly related to high PD-L1 levels (27). Perhaps most importantly, a significant correlation between IDO2 high expression and poor NSCLC prognosis was detected (27). Intriguingly, IDO2 expression was mainly associated with the basolateral side of the tumor cell membrane, and only few cells stained for IDO2 in the cytosol or nucleus. Therefore, these data would suggest a “membrane-associated” function, which may be distinct from the catalytic activity, similarly to IDO1 (12). Alternatively, the nuclear topology may further suggest a gene modulatory function by IDO2. In this regard, it is interesting to note that a previous study indicated that the nuclear-associated staining of IDO2 in the liver of conventional mice does not correlate with any difference in Trp/Kyn levels (38), thus possibly excluding the catalytic activity in nuclear-associated IDO2.

To evaluate the contribution of genetic variation in *IDO2* to the risk of NSCLC, we examined the frequencies of the two common SNPs in IDO2 as described above, namely rs10109853 (R248W) and rs4503083 (Y359X). By resorting to a cohort involving 145 NSCLC patients and 395 healthy matched controls, we found that the R248W displays a significantly different genotype distribution between NSCLC patients and controls, with the genotypes that include the minor allele conferring almost a 2-fold increased risk of NSCLC (**Table 1**). The Y359X SNP instead displayed only a trend towards association with NSCLC and only when using a dominant genetic model. Taken together, these results highlighted genetic variation in IDO2 as a key determinant of susceptibility to NSCLC. However, the IDO2 SNPs’ role appears to be distinct in NSCLC as compared to PDAC. In fact, whereas the presence of homo- or heterozygosity for the two SNPs increases the risk for NSCLC, the same condition will protect from PDAC.

**Table 1:** Association test results of *IDO2* genotypes and the risk of non-small-cell lung cancer (NSCLC).

Ref SNP	Genotypes	Controls (N=395)		NSCLC (N=145)	P-value
		n (%)	n (%)	OR (95% CI)	
	T/T	251 (63.5)	79 (54.9)	Reference	
rs4503083	T/A	131 (33.2)	56 (38.9)	1.36 (0.91 – 2.03)	0.145
(Y359X)	A/A	13 (3.3)	9 (6.3)	2.20 (0.91 – 5.34)	0.123
	T/A+A/A	144 (36.5)	65 (45.1)	1.43 (0.97 – 2.11)	0.073
	T/T	118 (29.9)	28 (19.3)	Reference	
rs10109853	T/C	192 (48.6)	88 (60.7)	1.93 (1.19 – 3.13)	**0.008**
(R248W)	C/C	85 (21.5)	29 (20.0)	1.44 (0.80 – 2.59)	0.231
	T/C+C/C	277 (70.1)	117 (80.9)	1.78 (1.12 – 2.84)	**0.016**

SNP, single nucleotide polymorphism; NSCLC, non-small-cell lung cancer; OR, odds ratio. One out of the 145 NSCLC patients had a missing genotype for rs4503083. Significant values are in bold.

**Figure 1:**
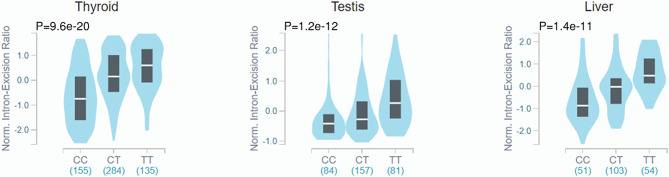
Violin plots of intron-excision ratios across different human tissues according to rs10109853 genotypes in *IDO2* (variant chr8_40005362_C_T_b38). Data were retrieved from the Genotype-Tissue Expression database (GTEx Analysis Release v8). The colored region indicates the density distribution of the samples in each genotype. The white line in the box plot indicates the median value of the intron excision ratio for each genotype.

Because the R248W SNP is described to impair IDO2 catalytic activity and the Y359X SNP generates a premature stop codon abolishing activity completely (26), the link observed between both SNPs and the development of NSCLC would support a relevant contribution of a defective enzymatic activity of IDO2 to disease pathogenesis. However, there are additional putative functional consequences of the *IDO2* SNPs worth considering. For example, while it does not affect gene expression, R248W is described to act as a strong splicing quantitative trait locus (sQTL) of *IDO2* across several tissues, but not the lung, in the Genotype-Tissue Expression (GTEx) project (**Figure 1**). Although the role of splicing events in IDO2 function remains unclear, the fact that the risk allele of R248W is reported to influence the intron-excision ratio of *IDO2* suggests an effect on transcript diversity that may also help explain its stronger association with NSCLC.

## Conclusion and Perspectives

IDO2 is a protein molecule that may represent an important drug target in cancer immunotherapy. In fact, IDO2 has some Trp catabolic activity, a catalytic function that, in the case of IDO1, has been demonstrated to be responsible for immunoregulatory effects. However, IDO2 is a very poor producer of Kyn and, consequently, Trp catabolic activity by this enzyme can very unlikely account for IDO2 biologic effects. In this regard, IDO2 may represent a sort of “pseudoenzyme”, i.e., a protein that is evolutionarily related to active enzymes, but lacks relevant catalytic activity (**Table 2**) (39, 40). Interestingly, the biological meaning of pseudoenzymes is currently the focus of intense research (41). Some authors suggested that the IDO2 catalytic function may depend on factors (42) whose identity has not been entirely identified yet and, therefore, it may have a better performance in “certain” *in vivo* conditions. However, as this is a hypothesis, the use of IDO2 catalytic inhibitors would be premature in cancer immunotherapy. An alternative hypothesis could in fact be that IDO2 preferentially uses substrates other than Trp and therefore a completely new story should be written for IDO2 as an enzyme. Disappointingly, no evidence for an alternative IDO2 catalytic activity has been provided so far. A great help in this regard may come from the crystallization of the IDO2 protein, which, unfortunately, has not been obtained yet.

**Table 2:** Main structural and functional features of human IDO1 and IDO2.

	Features	hIDO1	hID02	References
Structural	Trp metabolizing activity	K_cat_ (s^-1^) 2.97 ± 0.20	K_cat_ (s^-1^) 0.1 03 ± 0.006	(17)
		K_m_ (µM) 20.90 ± 3.95	K_m_ (µM) 6,809 ± 9 1 7	
	Presence of signaling motifs and their function	VPYCQL (ITIM1) Signaling activity	Absent	(10, 11)
		VYEGF (ITIM2) Signaling activity, protein degradation	MYEGV (putative ITIM) Unknown	
		YENM Pl3K binding	Absent	(12)
Functional	Frequency of expression in tumors	High	Low	(13, 24, 26, 27)
	Type of tumors	Endometrial, cervical, renal, gastric, and colorectal carcimonas Glioblastoma	Renal, gastric, and colorectal carcimonas Pancreas (PDAC), lung (NSCLC carcinomas)	(13, 24, 26, 27)
	Function in tumors	Immune escape	Not well defined, may be dependent on the genotype	(9, 14, 29)

The fact that IDO2 biology is still far from being understood also derives from the observations that this molecule appears to play opposite functions in both autoimmune and neoplastic diseases. In fact, in mouse experimental models of autoimmunity/chronic inflammation, IDO2 is pathogenetic in arthritis (20, 43) and protective in psoriasis (22). In humans, the presence of an *IDO2*-deficient functional status exerts protective effects in PDAC (29) but increases the risk of developing NSCLC (this study). As a whole, these data would suggest that IDO2 plays a context-dependent effect. In other words, the presence of cell- or microenvironment-specific factors as well as the direct interaction with specific protein partners would dictate the outcome of IDO2-associated effects. In this regard, the determination of the IDO2 interactome may be of great help. Unfortunately, at this time, we just know which known IDO1 partners do not interact with IDO2 (**Table 2**). These include SHP-1 and SHP-2 phosphatases that interact with IDO1 ITIM1 (mediating immunoregulatory IDO1 signaling activity in DCs (10); i.e., absent in IDO2) and class I PI3Ks that bind IDO1 *via* the YENM motif (mediating early endosome localization and thus the signaling function of IDO1 in DCs and tumor cell transfectants (12); also absent in IDO2). The ITIM2 motif is instead present in both mouse and human IDO2, but its role is still unknown. Interestingly, a recent study indicated that, upon treatment with lipopolysaccharide (LPS), IDO2 exerts negative regulatory effects on the IL-6 signaling pathway by reducing STAT3 expression in macrophages and possibly in other cell types *in vivo* (44). Notably, this effect occurred without changes in Kyn levels (44) and therefore it could be of much interest to clarify the molecular mode of action of IDO2 in this context.

In human neoplasia, although studies in only two types of tumor have been performed, a relevant issue appears to be the *IDO2* genotype, whose analysis could provide a valuable biomarker for informing treatment decisions (29). However, even in conditions such as PDAC in which the IDO2 loss-of-function seems to be protective, the fact that we do not know the true function/s of IDO2 in distinct cells and cellular microenvironments may have important consequences. As just an example, the use of IDO2 catalytic inhibitors may induce effects also in immune cells that should mount an effective anti-tumor response in neoplastic patients. Moreover, given the lack of information of a validated function, allocation of distinct profiles of IDO2 expression to the identified molecular subtypes of PDAC (45) cannot be performed yet.

In conclusion, although compounds that simultaneously inhibit IDO1 and IDO2 have already been identified (46), we believe that there is still a long road ahead before drug targeting of IDO2 can be effectively and safely used in cancer immunotherapy.

## Data Availability Statement

The raw data supporting the conclusions of this article will be made available by the authors, without undue reservation.

## Ethics Statement

The studies involving human participants were reviewed and approved by Comitato Etico Regionale dell’Umbria. The patients/participants provided their written informed consent to participate in this study.

## Author Contributions

All authors contributed to the article and approved the submitted version.

## Funding

This work was supported by Associazione Italiana per la Ricerca sul Cancro (AIRC 2019-23084; to UG) and the Italian Ministry of Education, University, and Research (PRIN 20173EAZ2Z; to CV). AC was supported by the Fundação para a Ciência e a Tecnologia (FCT) (UIDB/50026/2020, UIDP/50026/2020, CEECIND/03628/2017 and PTDC/MED-GEN/28778/2017) and the Northern Portugal Regional Operational Programme (NORTE 2020), under the Portugal 2020 Partnership Agreement, through the European Regional Development Fund (ERDF) (NORTE-01-0145-FEDER-000039).

## Conflict of Interest

The authors declare that the research was conducted in the absence of any commercial or financial relationships that could be construed as a potential conflict of interest.
